# *SLCO1A2, SLCO1B1* and *SLCO2B1* polymorphisms influences chloroquine and primaquine treatment in *Plasmodium vivax* malaria

**DOI:** 10.2217/pgs-2017-0077

**Published:** 2017-10-04

**Authors:** Vinicius A Sortica, Juliana D Lindenau, Maristela G Cunha, Maria Deise O Ohnishi, Ana Maria R Ventura, Ândrea KC Ribeiro-dos-Santos, Sidney EB Santos, Luciano SP Guimarães, Mara H Hutz

**Affiliations:** 1 Departamento de Genética, Universidade Federal do Rio Grande do Sul, Porto Alegre, RS, Brazil; 2 Laboratório de Microbiologia e Imunologia, Universidade Federal do Para, Belém, PA, Brazil; 3 Programa de Ensaios Clínicos em Malária, Instituto Evandro Chagas, Sistema de Vigilância Sanitária, Ministério da Saúde, Ananindeua, PA, Brazil; 4 Laboratório de Genética Humana e Médica, Universidade Federal do Pará, Belém, PA, Brazil; 5 Unidade de Bioestatística, Grupo de Pesquisa e Pós Graduação, Hospital de Clínicas de Porto Alegre, Porto Alegre, RS, Brazil

**Keywords:** chloroquine and primaquine, malaria, transporter genes

## Abstract

**Aim:**

The association of transporters gene polymorphisms with chloroquine/primaquine malaria treatment response was investigated in a Brazilian population.

**Patients & methods:**

Totally, 164 *Plasmodium vivax* malaria infected patients were included. Generalized estimating equations were performed to determine gene influences on parasitemia and/or gametocytemia clearance over treatment time.

**Results:**

Significant interaction between *SLCO2B1* genotypes and treatment over time for parasitemia clearance rate on day 2 were observed (*p*_FDR_ = 0.002). *SLCO1A2* and *SLCO1B1* gene treatment over time interactions were associated with gametocytemia clearance rate (*p*_FDR_ = 0.018 and *p*_FDR_ = 0.024). *ABCB1, ABCC4* and *SLCO1B3* were not associated with treatment response.

**Conclusion:**

The present work presents the first pharmacogenetic report of an association between chloroquine/primaquine responses with OATP transporters.

First draft submitted: 2 May 2017; Accepted for publication: 12 July 2017; Published online: 4 October 2017

*Plasmodium vivax* is the major cause of malaria outside Africa and represents a real challenge for malaria eradication in Asia and America continents, because of its intrinsic characteristic to develop dormant hypnozoite forms in the liver that cause subsequent infections in the blood [1–3].

In Brazil, chloroquine (CQ) and primaquine (PQ) combined therapy is the first choice treatment protocol for uncomplicated *P. vivax* malaria [4,5]. This treatment protocol with quinine derivatives combined targets asexual schizonts in blood and tissues, sexual gametocytes in blood and also hypnozoites in the liver [6–8]. Interindividual variability in CQ and PQ concentrations were reported in different populations [9–16]. Recently, the association of *CYP450* gene variants with CQ/PQ treatment and relapses were reported [17,18]; however, the effect of genetic variants in membrane transporters were not tested so far.

The potential role of drug transporters in antimalarial treatment became clear after the observation that malaria parasite expresses transporter genes in its digestive vacuole as defense mechanism against, for example, CQ. It is likely that there is a large overlap in substrate specificity between drug transporters in *Plasmodium* spp. and humans, therefore, genetic variants of drug transporters in humans might foster the development of drug resistance by, for example, lowering drug concentrations in red blood cells [19].

Transporters are integral membrane proteins that mediate the translocation of chemicals into and out of cells using active and passive mechanisms. ATP-binding cassette (ABC) and solute carrier (SLC) transporter families are formed by influx and efflux transporters expressed on membranes of polarized cells and have been shown to significantly affect concentrations of drugs in plasma and peripheral tissues, thus affecting drug efficacy and toxicity [20–22]. Membrane transporters such as MDR1 and multidrug proteins (MRP) are members of the ABC family and use ATP to move substrates across membranes [21,22]. Instead, the organic anion-transporting polypeptides (OATPs) move substrates against a concentration gradient without ATP expenditure [20], and together with ABC transporters are responsible for transport and availability of several endogenous and exogenous compounds. CQ seems to be an ATP-binding transporters inhibitor; however, some evidences indicate that this drug is a potential substrate for these transporters. The MRP transport system is responsible for CQ cellular direct efflux in multidrug-resistant tumor cells [23,24], and this drug could also act as substrate or inhibitor of human MDR1 transporter [25,26]. PQ also inhibits MDR1 and MRP1 drug transport without being a substrate [26]. Recently, CQ was described as an important inhibitor of OATP1A2 and OATP1B1 functions, representing a possible role in drug–drug interactions and malaria treatment [27,28].

To improve the understanding of how genetic variants influence CQ/PQ malaria treatment, the present study aims to investigate if *ABCB1, ABCC4, SLCO1A2, SLCO1B1, SLCO1B3* and *SLCO2B1* polymorphisms are associated with *P. vivax* malaria treatment response in a Brazilian population.

## Patients & methods

### Study population

The study population was composed of 164 *P. vivax* malaria patients. All subjects were born in Para state. This big Brazilian Amazonian state presents different risk of infection and transmission among different regions and cities [29]. Some patients were infected in their home cities whereas others while traveling to endemic regions. All subjects were diagnosed and treated in Belém at the Evandro Chagas Institute between 2007 and 2009. Sample and collection procedures were previously described [17]. Briefly, patients were clinically examined and received the standard treatment of 1500 mg of CQ associated with 210 mg of PQ in short regimen during a week (first day CQ 600 mg and PQ 30 mg, second and third days CQ 450 mg and PQ 30 mg, and last 4 days PQ 30 mg) as recommended by the Brazilian health authorities [30]. During the week treatment, patient response was daily accompanied by clinical examinations. Parasitemia and gametocytemia were daily estimated (density per microliter of blood by counting the number of parasites per 100 fields and double-checked blindly by two expert microscopists). Parasite counts were obtained before treatment and in the next 7 days of treatment. All subjects provided their written informed consent to participate in this study. Participants younger than 18 years (n = 17) had the informed consents signed by parents to participate in the study. The Ethics Committees of the Evandro Chagas Institute and Federal University of Pará approved the study protocol (CEP/IEC-0035 and CEP-CCS/UFPA 061/07).

### Genotyping

Genomic DNA was extracted from peripheral blood leukocytes using proteinase K digestion and standard phenol–chloroform procedures [31]. SNPs in *ABCB1, ABCC4, SLCO1A2, SLCO1B1, SLCO1B3* and *SLCO2B1* genes were determined by allelic discrimination with Taqman 5′-nuclease assays (Supplementary Table 1; real-time PCR, Applied Biosystems, CA, USA) according to the manufacturer's recommended protocol.

### Statistical analysis

Allele and genotype frequencies were estimated by gene counting. Deviation from Hardy–Weinberg equilibrium was verified by χ^2^ with Bonferroni correction. Haplotypes and linkage disequilibrium were estimated with PHASE 2.1.1 [32]. The individual proportions of European, African and Amerindian genetic ancestry from the study population were estimated as previously described [33]. Generalized estimating equation (GEE) is a repeated measure analysis focused on average changes in response over time and the impact of covariates on these changes. This method models the mean response as a linear function of covariates of interest via transformation or link function and can be used in studies in which data are asymmetric or the data distribution is difficult to verify due to small sample size [34]. This analysis was performed to determine the genetic influence in parasitemia or gametocytemia clearance by treatment over time considering a Gaussian distribution with an identity link function and an exchangeable correlation matrix structure. Parasitemia and gametocytemia levels were log-transformed before analysis due to their skewed distribution, but back-transformed values are presented in the results as geometric means. Age, gender, co-medication, parasitemia baseline level, gametocytemia baseline level and African and Amerindian genetic ancestry entered in models as covariates based on conceptual analyses of the literature and/or by means of a statistical definition (association with the study factor and with the outcome at p ≤ 0.15). Based on previous investigations with the same sample, *CYP2C8* genotypes were also used as a covariate in the gametocytemia analyses [17]. GEE analysis was performed with the SPSS18.0 (IBM Company, NY, USA) statistical package for Windows^®^. Benjamini–Hochberg procedure (false discovery rate [FDR]) for multiple comparisons was performed to control for multiple testing and corrected p-values were presented. Statistical significance was defined as a two-tailed p-value <0.05. To determine the effect sizes, Cohen's *d* test was calculated based on standardized differences between means [35].

## Results

Major demographic and clinical features from the study population are summarized in Table 1. Malaria patients were aged between 12 and 88 years (36.0 ± 15.6 years), and 29 patients (17.6%) used other medications in combination with CQ/PQ treatment to manage malaria symptoms or pre-existing diseases. All patients completed the 7 days treatment and adverse drug reactions were not reported. After complete treatment all patients presented negative results for parasites and gametocytes in blood and were monthly followed for 6 months. Twenty-seven patients (16.5%) presented relapses after at least 1 month of treatment and repeated the therapeutic regimen with no more relapses until the end of the follow-up period.

**Table 1.** **Study group main characteristics.**

**Characteristics**	**Malaria patients**
n	164

Age	36.0 (15.6)

Gender (male %)	68.5

Baseline parasitemia (parasites/μl)	8554.35 (50–75,000)

Baseline gametocytemia (gametocytes/μl)	110.93 (0–4500)

**Genetic ancestry**	

African	0.243 (0.09)

European	0.415 (0.11)

Native American	0.340 (0.12)

**Co-medication (%)**	

Antiemetic	4.2

Antipyretic/analgesic	12.2

Antacid	2.4

Antibiotic	1.2

Anthelmintic	0.6

Anticonvulsant	0.6

ACE inhibitor	0.6

Values for age and genetic ancestry are expressed as mean (SD).

Values for parasitemia and gametocytemia are expressed as median (range).

SD: Standard deviation.

*ABCB1, ABCC4, SLCO1A2, SLCO1B1, SLCO1B3* and *SLCO2B1* allele and genotype frequencies are shown in Supplementary Table 2. Haplotype frequencies are shown in Supplementary Table 3. Genotype distributions did not deviate significantly from Hardy–Weinberg equilibrium in the study population. *ABCB1, SLCO1B1* and *SLCO1B3* allelic, genotype and haplotype frequencies showed no statistical differences from frequencies observed in a general population sample from Belém (data not shown, but available upon request) [36].

### Influence of ABCB1 & SLCO2B1 in parasitemia clearance

*ABCB1* and *SLCO2B1* genes were associated with the clearance rate over treatment time in the model adjusting for age, gender, co-medication, parasitemia baseline level and genetic ancestry. After FDR procedure, *ABCB1* gene was no longer associated with parasitemia clearance rate over treatment time. No main gene effect was observed for both genes (Table 2).

**Table 2.** **Mean parasitemia over treatment time according to *ABCB1* and *SLCO2B1* genes.**

**Gene**	**Treatment**	**Mean parasitemia (parasites/μl)**			**p**		***p_FDR_***		***d*^†^**
					**Gene**	Gene*time	**Gene**	Gene*time	
*ABCB*^‡^		CGC/CGC (n = 43)	TnonGT (n = 83)	Others (n = 31)					
	Day 0	3894.85	3574.30	3680.04					
		(3211.5–4723.6)	(2728.8–4681.8)	(2729–4962.5)					
	Day 1	360.53	244.93	279.32	0.568	0.044	NS	0.132	0.41
		(186.23–697.95)	(141.20–424.86)	(153.34–508.80)					
	Day 2	0.44	0.78	1.98					
		(0.12–1.67)	(0.29–2.10)	(0.55–7.12)					
	Day 3	0.01	0.02	0.01					
		(0.005–0.014)	(0.012–0.048)	(0.004–0.025)					

*SLCO2B1*^§^		AA (n = 26)	AG (n = 79)	GG (n = 50)					
	Day 0	3626.79	3722.19	3758.23					
		(2734.5–4810.2)	(2879.6–4811.3)	(2809.1–5028.0)					
	Day 1	321.52	339.01	201.12	0.073	<0.0002	NS	0.002	0.89
		(163.85–630.90)	(225.43–509.82)	(82.73–488.94)					
	Day 2	0.10	0.86	3.23					
		(0.02–0.48)	(0.33–2.28)	(0.99–10.54)					
	Day 3	0.01	0.02	0.01					
		(0.004–0.018)	(0.011–0.036)	(0.005–0.030)					

Mean parasitemia adjusted for age, gender, genetic ancestry, co-medication and parasitemia baseline level.

Parasitemia is expressed as geometric mean (95% CI).

^†^Effect size Cohen's *d*.

^‡^Haplotypes based on SNPs 3435C>T, 2677G>A/T and 1236C>T.

^§^Genotypes from SNP 935G>A.

NS: Not significant.

**Figure 1.**
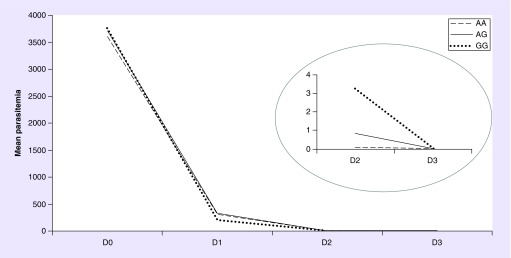
**Mean parasitemia clearance during chloroquine/primaquine regimen according to *SLCO2B1* genotypes.** Generalized estimating equations method with age, gender, co-medication, parasitemia baseline level and genetic ancestry as covariates; gene*time *p_FDR_* = 0.002 and *d* = 0.89. In the right circle, it was highlighted the mean parasitemia clearance in the days 2 and 3. On day 2, AA genotype showed a higher parasitemia clearance rate over treatment time than GG genotypes. Mean parasitemia means are presented as parasites/ul (for details, see ‘Patients & methods’ section).

The model including treatment over time with *SLCO2B1* genotypes is represented in Figure 1. Although no main genotype effect was observed (p_FDR_ = 0.100), a significant interaction effect between genotypes and treatment over time for parasitemia clearance rate during treatment (p_FDR_ = 0.002) was observed. On day 2 *SLCO2B1* AA carriers showed an interaction effect between genotypes and parasitemia clearance rate over treatment time when compared with GG genotype carriers (AA vs AG: p = 0.076; AA vs GG: p = 0.002; and AG vs GG: p = 0.262). Considering Cohen's classification, this interaction presents a large effect size (*d* = 0.89) [37].

*ABCC4, SLCO1A2, SLCO1B1* and *SLCO1B3* variants were not associated with parasitemia clearance rate.

### Influence of SLCO1A2 & SLCO1B1 in gametocytemia clearance

**Figure 2.**
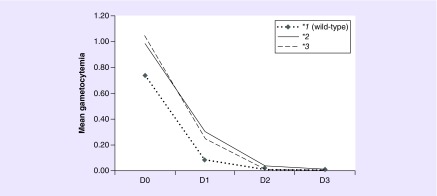
**Mean gametocytemia clearance during chloroquine/primaquine regimen according to *SLCO1A2* haplotypes.** Generalized estimating equations method with age, gender, co-medication, gametocytemia baseline level, *CYP2C8* genotypes and genetic ancestry as covariates. On day 2 *SLCO2B1* AA carriers showed an interaction effect between genotypes and parasitemia clearance rate over treatment time when compared with GG genotype carriers (AA vs AG: p = 0.076; AA vs GG: p = 0.002; and AG vs GG: p = 0.262). Gene*time *p_FDR_* = 0.018 and *d* = 0.59. Gametocytemia means are presented as gametocytes/ul (for details, see ‘Patients & methods’ section).

*SLCO1A2* and *SLCO1B1* genes were associated with the gametocytemia clearance rate over treatment time in GEE analyses adjusted for age, gender, co-medication, parasitemia baseline level, *CYP2C8* genotypes and genetic ancestry. *SLCO1A2* was associated with gametocytemia (p = 0.003); SLCO1A2*2 carriers presented higher mean gametocytemia SLCO1A2*1 homozygotes (p = 0.002). The model including treatment over time in the presence of *SLCO1A2* genotypes is represented in Figure 2; a significant interaction effect between genotypes and treatment over time for gametocytemia clearance rate during treatment was observed (p_FDR_ = 0.018). The model showed that SLCO1A2*2 and SLCO1A2*3 carriers have clearance of gametocytes at a lower rate as compared with SLCO1A2*1 homozygotes during treatment. Following effect size Cohen's interpretation scale, *SLCO1A2* model showed medium effect size (*d* = 0.59).

**Figure 3.**
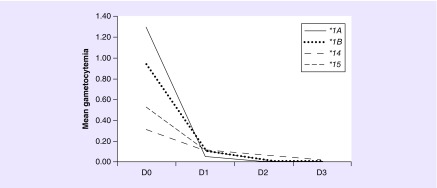
**Mean gametocytemia clearance during chloroquine/primaquine regimen according to *SLCO1B1* haplotypes.** Generalized estimating equations method with age, gender, co-medication, gametocytemia baseline level, *CYP2C8* genotypes and genetic ancestry as covariates; gene*time *p_FDR_* = 0.024 and *d* = 1.20. A lower gametocytemia clearance rate over time by SLCO1B1*14 carriers compared with SLCO1B1*1a and SLCO1*1b allele carriers is represented (on second day: *1a vs *14; p = 0.001 and *1b vs *14; p = 0.002). Gametocytemia means are presented as gametocytes/ul (for details, see ‘Patients & methods’ section).

The main effect of *SLCO1B1* genotype was not associated with mean gametocytemia in the analysis (p = 0.086), nevertheless, a significant interaction effect between genotype and gametocytemia clearance rate over time (p_FDR_ = 0.024) was observed. The model represented in Figure 3 showed lower gametocytemia clearance rate over time by SLCO1B1*14 carriers compared with SLCO1B1*1a and SLCO1*1b allele carriers (on second day: *1a vs *14; p = 0.001 and *1b vs *14; p = 0.002; Figure 3). This model showed large effect sizes (*d* = 1.20) based on Cohen's interpretation scale [37].

Models including *ABCB1, ABCC4, SLCO1B2* and *SLCO1B3* were not associated with gametocytemia clearance rate.

## Discussion

The main finding of the present study was that SLC transporters influence *P. vivax* malaria treatment in a Brazilian Amazonian population. CQ and PQ have a synergistic effect as schizonticide, gametocide and hipnozontocide, and could be either substrates or inhibitors for these transporters [15,19,20].

Several studies showed fundamental differences in the pharmacodynamics and pharmacokinetic action of CQ between *Plasmodium vivax* and *P. falciparum* [38,39]. Moreover, CQ resistance grades in *P. vivax* are not fully understood as it is for *P. falciparum*. The patterns observed in *in vitro* tests for resistance did not show relapses or recrudescence before 28 days [8,40]. Current *P. vivax in vivo* studies are unable to distinguish a recrudescence of CQ-resistant parasites from a relapse or a new infection [41].

Knowledge about the mechanisms and molecular markers of CQ resistance in *P. vivax* remains limited. Due to intrinsic biological dissimilarities, extrapolation from *P. falciparum* may not reflect the true drug resistance mechanisms of *P. vivax*, thus limiting the number of parasite markers definitively identified in *P. vivax* [42]. Although how the interactions among CQ and PQ with OATP1A2, OATP1B1 and OATP2B1 occur is not fully understood, the results of the present study suggest that polymorphisms in these transporters should alter the availability of both drugs in different tissues interfering with their action in a specific manner affecting the different sexual forms of *P. vivax*. Thus, it is an important variability factor that should be considered together with the metabolism variability of CQ and PQ on treatment response.

SLCs’ organic anion-transporting polypeptides (OATPs) are plasma membrane proteins that mediate the active cellular influx of a variety of amphipathic compounds. These proteins were expressed in apical and basolateral membranes of polarized cells in tissues such as liver and kidneys, as well as in the intestinal wall and the blood–brain barrier, and may affect pharmacokinetics and effects of their substrates [43,44].

The nonsynonymous 935G>A SNP in *SLCO2B1* was associated with parasitemia clearance rate in malaria treatment in this study population. Homozygous patients for the 935A allele showed a significant higher parasitemia clearance rate over treatment time when compared with GG homozygotes. OATP2B1 also is expressed in the sinusoidal membrane of hepatocytes in the luminal membrane of small intestinal enterocytes, suggesting that it participate in drugs uptake from blood and absorption [45,46]. Recently, the expression of this transporter on red blood cell membrane was described, but the action of antimalarial drugs such as CQ as inhibitor or substrate of OATP2B1 was not observed *in vitro* [28]. Despite this, 935G>A SNP showed an important effect in CQ/PQ treatment variability in the present study and pharmacokinetic mechanisms should be further investigated.

OATP1B1 is mainly expressed in the sinusoidal membrane of human hepatocytes diffusely distributed in the liver lobulus [44]. This protein mediates uptake of its substrates from blood into the liver [45] and is encoded by *SLCO1B1* gene. Polymorphisms in *SLCO1B1* were well characterized and associated with statins pharmacogenetics [47–52]. In the present study, the SLCO1B1*14 allele carriers have lower gametocytemia clearance rate over treatment time in malaria. This allele is formed by 388G and 463A SNP variants, which were associated with reduced transport activity *in vitro* [53]. Recently, it has been shown that CQ increases OATP1B1 levels in different models *in vitro* and interacts with this transporter as an inhibitor decreasing transport activity [27].

OATP1A2 is highly expressed in the brain, but it is also expressed in, intestine, kidneys, lung and testes in different amounts [54–56]. OATP1A2 is encoded by *SLCO1A2* gene, and its polymorphisms were mainly associated with cancer treatment pharmacokinetics [57,58]. The results of the present work showed that SLCO1A2*2 and SLCO1A2*3 presented lower gametocytes clearance rates over treatment time. These alleles are associated with changes in the protein sequence (I13T and E172D), and are related to an increased and decreased uptake of substrates, respectively [59]. Recently, the expression of this transporter on red blood cell membrane was described as well as the quinine transport activity inside these cells [28]. CQ is an important OATP1A2 inhibitor [28], therefore, the influence of OATP1A2 in CQ and PQ treatment should be better investigated.

The present study also showed the association of *ABCB1* gene haplotypes on parasitemia clearance rate over treatment time. MDR1 (or P-glycoprotein) is responsible for the active efflux of many drugs, by biliary and renal excretion [20,21,60,61]. The present results showed that *ABCB1* T/nonG/T haplotype carriers (3435C>T, 2677G>A/T and 1236C>T) showed a lower parasitemia clearance rate over treatment time when compared with wild-type haplotype CGC homozygotes; however, after the FDR procedure, this difference was no longer statistically significant. CQ seems to be a broad inhibitor of ABC transporters and a potential substrate of some ABCs. The significance loss in the analysis after multiple comparisons correction could be related to the polymorphism small effect size. The role of *ABCB1* variants on CQ pharmacokinetics in malaria treatment should be better investigated in future studies.

The observational-naturalistic design of our study, moderate sample size and the absence of CQ/PQ plasma levels information in our patients are limitations of the present study. However, this design might be valuable to better appreciate the role of genetic factors in routine clinical practice beyond the realm of controlled clinical trials, but some caveats of this kind of studies should be considered. First, we had no internal control to correct for any effect of time (e.g., regression to the mean) or expectancy bias because we did not have a placebo arm in this trial. Second, we did not control for parasite resistance. Nevertheless, CQ-resistant *P. vivax* has been estimated to vary from 4.4 to 10% in the Amazonian region [62] and all patients presented negative results after treatment. The role of transporter gene variability is associated with the rate of clearance and not with efficacy *per se*. Although it is not possible to exclude that the effects we observed were due to lack of adherence to treatment, there is no reason to expect a preferential compliance to CQ/PQ treatment according to transporter gene genotypes. This study design also did not allow to evaluate PQ effect in liver hypnozoites, or to evaluate the effect of transporter genes in relapses occurrence.

## Conclusion

The present study reports important effects of transporters gene variants in CQ/PQ pharmacokinetics, which could represent an important factor to permit *P. vivax* resistance to this treatment. The study was the first to describe these pharmacogenetic influences in *P. vivax* malaria treatment and the results found should be replicated in larger and independent samples.

## Future perspective

Despite all the efforts to develop a multiple drug therapy that has a good response in the malaria treatment, this disease still is an important morbidity and mortality factor in several world regions, among them the Amazonian region. For now, pharmacogenetic studies in this kind of disease are scarce, mainly because of their complexity. However, the present study reports an important contribution to the development of the personalized treatment in the malaria disease. More studies analyzing the role of the genetic background in the drug response could help in better drug therapy prescription, minimizing their adverse effects and improving their effectiveness.

**Summary points**

- To improve the understanding of how genetic variants influence chloroquine/primaquine malaria treatment, the present study investigated if *ABCB1, ABCC4, SLCO1A2, SLCO1B1, SLCO1B3* and *SLCO2B1* polymorphisms are associated with *Plasmodium vivax* malaria treatment response.
- Analyses of the effect of different genotypes on treatment efficacy were performed using generalized estimating equations to determine the genetic influence on parasitemia or gametocytemia clearance over time.
- *ABCB1* and *SLCO2B1* genes were associated with the parasitemia rate over treatment time in the model adjusting for age, gender, co-medication, parasitemia baseline level and genetic ancestry. After FDR procedure, *ABCB1* gene was no longer associated with parasitemia rates.
- A significant interaction effect between *SLCO2B1* genotypes and treatment over time for parasitemia elimination rates during treatment (p_FDR_ = 0.002) was observed. On day two *SLCO2B1* AA carriers showed higher parasitemia elimination rates than GG genotype carriers (AA vs AG: p = 0.076; AA vs GG: p = 0.002; and AG vs GG: p = 0.262). Considering Cohen's classification, this interaction presents a large effect size (*d* = 0.89).
- *SLCO1A2* and *SLCO1B1* genes were associated with gametocytemia rate over treatment time in generalized estimating equation analyses adjusted for age, gender, co-medication, parasitemia baseline level, *CYP2C8* genotypes and genetic ancestry. *SLCO1A2* was associated with gametocytemia (p = 0.003).
- A significant interaction effect between genotypes and treatment over time for gametocytemia elimination rates during treatment was observed (p_FDR_ = 0.018). The model showed that SLCO1A2*2 and SLCO1A2*3 carriers eliminates gametocytes in a lower rate as compared with SLCO1A2*1 homozygotes during treatment.
- The main effect of *SLCO1B1* genotype was not associated with mean gametocytemia in the analysis (p = 0.086), nevertheless, a significant interaction effect between genotype and treatment over time (p_FDR_ = 0.024) was observed.
- SLCO1B1*14 carriers showed lower gametocytemia elimination rates compared with SLCO1B1*1a and SLCO1*1b allele carriers (on second day: *1a vs *14; p = 0.001 and *1b vs *14; p = 0.002). This model showed large effect sizes (*d* = 1.20) based on Cohen's interpretation scale.
