# Supplementary material for: SLCO1A2, SLCO1B1 and SLCO2B1 polymorphisms influences chloroquine and primaquine treatment in Plasmodium vivax malaria
Source: Pharmacogenomics. 2017 Oct 4;18(15):1393–400. doi: 10.2217/pgs-2017-0077 (PMC7099631; doi:10.2217/pgs-2017-0077)
Supplement: Supplementary file 1 [file pgs-18-1401-s1.docx]

**Supplemental Table 1** List of SNPs genotyped in the present study

| Gene | SNP | Effect | dbSNP ID | Assay ID |
| --- | --- | --- | --- | --- |
| *ABCB1* | 3435C>T | Synonymous | rs1045642 | C___7586657_20 |
|  | 2677G>A/T | S893T | rs2032582 | # |
|  | 1236C>T | Synonymous | rs1128503 | C___7586662_10 |
| *ABCC4* | 912G>T | K304N | rs2274407 | C__16181780_20 |
| *SLCO1A2* | 516A>C | E172D | rs11568563 | C__25605897_10 |
|  | 38T>C | I13T | rs10841795 | C__25605906_20 |
| *SLCO1B1* | 388A>G | N130D | rs2306283 | C___1901697_20 |
|  | 463C>A | P155T | rs11045819 | * |
|  | 521T>C | V174A | rs4149056 | C__30633906_10 |
| *SLCO1B3* | 334T>G | S112A | rs4149117 | C__25639181_40 |
|  | 699G>A | M233I | rs7311358 | C__25765587_40 |
| *SLCO2B1* | 935G>A | R290Q | rs12422149 | C___3101331_10 |

# Assay C_11711720D_40 for 2677G>A alleles and assay C_11711720C_30

for 2677G>T alleles.

*Custom assay

**Supplemental Table 2** Transporter genes allelic and genotypic frequencies

| Gene | SNP | dbSNP ID | N |  | Genotypes | | |  | Alleles | |
| --- | --- | --- | --- | --- | --- | --- | --- | --- | --- | --- |
| *ABCB1* | 3435C>T | rs1045642 |  |  | CC | CT | TT |  | C | T |
|  |  |  | 164 |  | 55 (33.5) | 72 (43.9) | 37 (22.6) |  | 182 (56.0) | 146 (44.0) |
|  | 2677G>A/T | rs2032582 |  |  | GG | G/NonG | NonG |  | G | NonG |
|  |  |  | 164 |  | 70 (42.7) | 74 (45.2) | 20 (12.1) |  | 214 (65.2) | 114 (34.8) |
|  | 1236C>T | rs1128503 |  |  | CC | CT | TT |  | C | T |
|  |  |  | 164 |  | 60 (36.6) | 74 (45.2) | 30 (18.2) |  | 194 (59.1) | 134 (40.9) |
| *ABCC4* | 912G>T | rs2274407 |  |  | CC | CA | AA |  | C | A |
|  |  |  | 164 |  | 131 (79.3) | 33 (20.1) | 1 (0.6) |  | 293 (89.4) | 35 (10.6) |
| *SLCO1A2* | 516A>C | rs11568563 |  |  | AA | AC | CC |  | A | C |
|  |  |  | 164 |  | 154 (93.9) | 10 (6.1) | 0 |  | 318 (97.0) | 10 (3.0) |
|  | 38T>C | rs10841795 |  |  | AA | AG | GG |  | A | G |
|  |  |  | 164 |  | 146 (89.0) | 17 (10.4) | 1 (0.6) |  | 309 (94.8) | 19 (5.8) |
| *SLCO1B1* | 388A>G | rs2306283 |  |  | AA | AG | GG |  | A | G |
|  |  |  | 164 |  | 32 (19.5) | 90 (54.9) | 42 (25.6) |  | 154 (46.9) | 174 (53.1) |
|  | 463C>A | rs11045819 |  |  | CC | CA | AA |  | C | A |
|  |  |  | 164 |  | 141 (86.0) | 22 (13.4) | 1 (0.6) |  | 304 (92.7) | 24 (7.3) |
|  | 521T>C | rs4149056 |  |  | TT | TC | CC |  | T | C |
|  |  |  | 164 |  | 5 (3.0) | 49 (29.9) | 110 (67.7) |  | 59 (17.9) | 269 (82.1) |
| *SLCO1B3* | 334T>G | rs4149117 |  |  | TT | TG | GG |  | T | G |
|  |  |  | 164 |  | 10 (6.2) | 65 (39.5) | 89 (54.3) |  | 85 (25.9) | 243 (74.1) |
|  | 699G>A | rs7311358 |  |  | GG | GA | AA |  | G | A |
|  |  |  | 164 |  | 10 (6.2) | 65 (39.5) | 89 (54.3) |  | 85 (25.9) | 243 (74.1) |
| *SLCO2B1* | 935G>A | rs12422149 |  |  | GG | GA | AA |  | G | A |
|  |  |  | 164 |  | 53 (32.3) | 84 (51.2) | 27 (16.6) |  | 190 (57.9) | 138 (42.1) |

**Supplemental Table 3** Transporters gene haplotype frequencies

| Gene | Haplotypes | | | | | N | Frequency (%) |
| --- | --- | --- | --- | --- | --- | --- | --- |
| *ABCB1* |  | 3435C>T | 2677G>A/T | 1236C>T |  |  |  |
|  |  | T | G | T |  | 15 | 4.6 |
|  |  | T | G | C |  | 25 | 7.7 |
|  |  | T | NonG | T |  | 101 | 30.7 |
|  |  | T | NonG | C |  | 5 | 1.5 |
|  |  | C | G | T |  | 15 | 4.6 |
|  |  | C | G | C |  | 160 | 48.8 |
|  |  | C | NonG | T |  | 3 | 0.9 |
|  |  | C | NonG | C |  | 4 | 1.2 |
|  |  |  |  |  |  |  |  |
| *SLCO1A2* |  | 516A>C | 38T>C |  |  |  |  |
| *1 |  | A | A |  |  | 298 | 91.0 |
| *2 |  | A | G |  |  | 19 | 5.9 |
| *3 |  | C | A |  |  | 10 | 3.1 |
|  |  |  |  |  |  |  |  |
| *SLCO1B1* |  | 388A>G | 463C>A | 521T>C |  |  |  |
| *1a |  | A | C | T |  | 150 | 45.7 |
| *5 |  | A | C | C |  | 4 | 1.2 |
| *1B |  | G | C | T |  | 95 | 29.0 |
| *15 |  | G | C | C |  | 55 | 16.8 |
| *14 |  | G | A | T |  | 24 | 7.3 |
|  |  |  |  |  |  |  |  |
| *SLCO1B3* |  | 334T>G | 699G>A |  |  |  |  |
|  |  | G | A |  |  | 243 | 74.1 |
|  |  | T | G |  |  | 85 | 25.9 |
